# Posterior reversible encephalopathy syndrome following immunoglobulin therapy in a patient with Miller-Fisher syndrome

**DOI:** 10.1590/0100-3984.2015.0129

**Published:** 2016

**Authors:** Bruno Niemeyer de Freitas Ribeiro, Tiago Medina Salata, Rafael Silveira Borges, Edson Marchiori

**Affiliations:** 1 Instituto Estadual do Cérebro Paulo Niemeyer, Rio de Janeiro, RJ, Brazil.; 2 Hospital Casa de Portugal / 3D Diagnóstico por Imagem, Rio de Janeiro, RJ, Brazil.; 3 Universidade Federal do Rio de Janeiro (UFRJ), Rio de Janeiro, RJ, Brazil.

*Dear Editor,*

A 54-year-old female patient presenting with ophthalmoparesis, ataxia and areflexia for one week. The patient denied fever, muscle weakness, and did not report any previous comorbidity. At physical examination, she was normotensive, oriented, with bilateral flexor cutaneous-plantar reflex and preserved superficial/deep sensitivity. Human immunodeficiency virus, Epstein-Barr virus, cytomegalovirus, HTLV-1 and VDRL serologies were negative. Considering such findings, the hypothesis of Miller-Fisher syndrome was raised, and liquor cerebrospinalis analysis demonstrated hyperproteinorachia, confirming the diagnosis.

Within 24-48 hours after immunoglobulin therapy initiation, the patient presented with intense headache followed by tonic-clonic seizures and later decreased level of consciousness, with no association with hypertensive peaks. Magnetic resonance imaging (MRI) (Figure 1A,B,C) showed sparse hyperintense areas in the white substance, bilaterally on T2-weighted and FLAIR sequences, predominantly in the parieto-occipital regions, without diffusion restriction and without gadolinium enhancement, demonstrating an imaging pattern suggestive of posterior reversible encephalopathy syndrome (PRES). After the therapy suspension and adoption of support measures, the patient progressed satisfactorily, with no sequelae and reversion of the MRI findings (Figure 1D).

**Figure 1:**
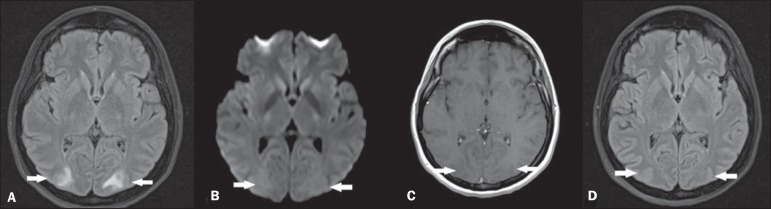
**A:** Axial MRI FLAIR sequence demonstrating hyperintensity in the occipital lobes white substance bilaterally and symmetrically (arrows). **B:** Axial diffusionweighted MRI does not demonstrate any alterations (arrows). **C:** Contrast-enhanced T1-weighted sequence revealing absence of gadolinium-enhanced areas (arrows). **D:** Axial FLAIR sequence acquired after four weeks demonstrating resolution of the alterations in the occipital lobes white substance (arrows).

The Brazilian radiological literature has recently highlighted the relevant role played by MRI in the improvement of the diagnosis of central nervous system conditions^(1-5)^.

PRES is a clinical-radiological entity of varied etiology, generally occurring in the setting of severe arterial hypertension. In some cases, however, it may be associated with immunosuppressive therapy, and is rarely described in the literature after the use of immunoglobulin^(6-12)^. Its physiopathogenesis is characterized by the presence of endothelial lesion and dysfunction of cerebral autoregulation mechanisms, leading to hypoperfusion and vasogenic edema^(7-12)^. The clinical manifestations present acute/ subacute onset characterized by headache, decreased level of consciousness, visual alterations, tonic-clonic seizures and focal neurological signs. The symptoms are progressive. Complete regression is achieved provided the syndrome is appropriately treated; otherwise irreversible damages may occur^(6-11)^.

MRI findings are quite suggestive and characterized by hyperintense areas on T2-weighted and FLAIR sequences, in general affecting the white substance bilaterally and symmetrically, with predilection for the parieto-occipital region. It may also affect the frontal lobes, internal and external capsules, cerebellum and encephalic trunk^(7-9)^. At early stages of the condition, diffusion MRI does not demonstrate any abnormalities, but inappropriate management may result in irreversible damages presented as diffusion restriction corresponding to cytotoxic edema.

Recent studies by means of retrospective analysis, utilizing MRI and laboratory data, have demonstrated the association between PRES and albumin serum levels. There are evidences that significantly decreased albumin serum levels lead to a higher risk to develop vasogenic-type edema^(12)^. This is due to the fact that, in conditions with endothelial damages caused by inflammatory processes, the decrease in the colloidosmotic pressure, directly related to the albumin levels, may facilitate the development of vasogenic edema. Thus, the early administration of human serum albumin might prevent ischemic damages and reduce possible sequelae^(12)^.

Finally, despite being rare after administration of immunoglobulin, PRES should be considered in cases where typical MRI findings are present. One should not wait until the onset of a hypertensive episode to take such a diagnostic possibility into consideration.

## References

[1] Bimbato EM, Carvalho AG, Reis F. (2015). Toxic and metabolic encephalopathies: iconographic
essay. Radiol Bras.

[2] Castro FD, Reis F, Guerra JGG. (2014). Intraventricular mass lesions at magnetic resonance imaging:
iconographic essay - part 1. Radiol Bras.

[3] Ono SE, Carvalho A, Gasparetto EL (2014). X-linked adrenoleukodystrophy: correlation between Loes score and
diffusion tensor imaging parameters. Radiol Bras.

[4] Alfenas R, Niemeyer B, Bahia PRV (2014). Parry-Romberg syndrome: findings in advanced magnetic resonance
imaging sequences - case report. Radiol Bras.

[5] Barbosa JHO, Santos AC, Salmon CEG. (2015). Susceptibility weighted imaging: differentiating between
calcification and hemosiderin. Radiol Bras.

[6] Stetefeld HR, Lehmann HC, Fink GR (2014). Posterior reversible encephalopathy syndrome and stroke after
intravenous immunoglobulin treatment in Miller-Fisher syndrome/Bickerstaff
brain stem encephalitis overlap syndrome. J Stroke Cerebrovasc Dis.

[7] McKinney AM, Short J, Truwit CL (2007). Posterior reversible encephalopathy syndrome: incidence of
atypical regions of involvement and imaging findings. AJR Am J Roentgenol.

[8] Pereira PR, Pinho J, Rodrigues M (2015). Clinical, imagiological and etiological spectrum of posterior
reversible encephalopathy syndrome. Arq Neuropsiquiatr.

[9] Bartysnki WS, Boardman JF. (2007). Distinct imaging patterns and lesion distribution in posterior
reversible encephalopathy syndrome. AJNR Am J Neuroradiol.

[10] Bartynski WS (2008). Posterior reversible encephalopathy syndrome, part 1: fundamental
imaging and clinical features. AJNR Am J Neuroradiol.

[11] Wada A, Yoshida R, Oda K (2005). Acute encephalopathy associated with intravenous immunoglobulin
therapy. AJNR Am J Neuroradiol.

[12] Pirker A, Kramer L, Voller B (2011). Type of edema in posterior reversible encephalopathy syndrome
depends on serum albumin levels: an MR imaging study in 28
patients. AJNR Am J Neuroradiol.

